# Oxidative Stress, Sperm DNA Fragmentation, or Both? Optimizing Test Selection in Male Infertility Evaluation

**DOI:** 10.3390/antiox15030293

**Published:** 2026-02-26

**Authors:** Aris Kaltsas, Stamatis Papaharitou, Pallav Sengupta, Ramadan Saleh, Ashok Agarwal

**Affiliations:** 1 Third Department of Urology, Attikon University Hospital, School of Medicine, National and Kapodistrian University of Athens, 12462 Athens, Greece; ares-kaltsas@hotmail.com; 2 Global Andrology Forum, Global Andrology Foundation, Moreland Hills, OH 44022, USA; sdrcauth@gmail.com (S.P.); pallav_cu@yahoo.com (P.S.); ramadan_saleh@med.sohag.edu.eg (R.S.); 3 Andrology Laboratory and Sperm Bank, 54622 Thessaloniki, Greece; 4 Department of Biomedical Sciences, College of Medicine, Gulf Medical University, Ajman P.O. Box 4184, United Arab Emirates; 5 Department of Dermatology, Venereology & Andrology, Faculty of Medicine, Sohag University, Sohag 82524, Egypt; 6 Ajyal IVF Center, Ajyal Hospital, Sohag 82511, Egypt; 7 Cleveland Clinic, Cleveland, OH 44195, USA

**Keywords:** 8-hydroxy-2′-deoxyguanosine, Comet assay, oxidation–reduction potential, oxidative stress, reactive oxygen species, recurrent pregnancy loss, Sperm Chromatin Structure Assay, sperm DNA fragmentation

## Abstract

Oxidative stress (OS) and sperm DNA fragmentation (SDF) are complementary contributors to male infertility. OS characterizes a compromised seminal redox status, whereas SDF quantifies downstream genomic damage. Human sperm are highly susceptible to redox damage due to lipid-rich membranes and disrupted post-meiotic DNA-repair capacity. Excess reactive oxygen species (ROS) can cause lipid peroxidation, oxidative base lesions, and DNA strand breaks that impair fertilization, embryo development, and pregnancy outcomes. This review explains how OS promotes genomic instability and summarizes the main laboratory assays that assess redox status and SDF in semen. These include direct ROS chemiluminescence assay, oxidation–reduction potential, total antioxidant capacity/ferric reducing antioxidant power, and lipid peroxidation biomarkers, alongside SDF platforms (Sperm Chromatin Structure Assay, terminal deoxynucleotidyl transferase dUTP nick-end labeling, alkaline/neutral Comet, and sperm chromatin dispersion). Additionally, guideline-aligned indications are highlighted to clarify the conditions for testing OS and SDF. OS testing is most relevant in men with leukocytospermia or suspected genital tract infection or inflammation, including dysbiosis; in cases of major modifiable exposures such as smoking or heat; and for early monitoring after treatment. SDF testing is particularly informative in couples with recurrent pregnancy loss and in unexplained infertility with normal semen parameters. Combined OS and SDF testing is recommended in clinical varicocele, repeated in vitro fertilization (IVF) or intracytoplasmic sperm injection (ICSI) failure, poor embryo development, and follow-up after targeted therapy. Management centers on treating infection and inflammation, improving lifestyle and environmental factors, considering varicocelectomy when indicated, using targeted antioxidant therapy in men with documented OS, and selectively applying sperm selection technologies or testicular sperm for ICSI when SDF remains high. Priorities include assay standardization, etiologic attribution of DNA damage, and trials testing OS/SDF-guided pathways with live birth as the primary endpoint. When used selectively and in the appropriate context, OS and SDF testing can help refine diagnosis, improve counseling, and help personalize care of infertile couples.

## 1. Introduction

Infertility affects an estimated 15–17% of couples worldwide; male factors contribute to roughly half of cases, and a sizeable fraction remains idiopathic after routine evaluation [1,2,3]. Conventional semen analysis captures concentration, motility, and morphology but incompletely explains functional defects such as chromatin instability, oxidative imbalance, and DNA damage. This has directed attention to seminal oxidative stress (OS) and sperm DNA fragmentation (SDF) which have emerged as key determinants in otherwise unexplained male infertility [4,5].

Human spermatozoa are susceptible to redox imbalance and oxidative SDF because of their lipid-rich membranes, residual histone content, protamine deficiencies and markedly limited DNA-repair capacity. Excess reactive oxygen species promote lipid peroxidation and oxidative DNA lesions, whereas SDF denotes structural compromise of the paternal genome associated with subfertility, impaired embryo development, and increased miscarriage risk [6,7]. Although inter-related, OS and SDF are not synonymous: oxidative injury can generate strand breaks, yet non-oxidative processes (e.g., defective chromatin packaging, abortive apoptosis or aberrant chromatin remodeling) also contribute. Accordingly, characterizing both the redox milieu and DNA integrity provides complementary information beyond routine semen metrics [8].

Despite methodological advances, uncertainties remain regarding clinical utility, standardization of thresholds, and how test results should meaningfully alter management pathways: which assays most reliably capture seminal redox status and DNA integrity; how pre-analytics and thresholds should be standardized across platforms; in which clinical scenarios testing improves diagnosis, prognosis, or management; and which mechanism-targeted interventions translate into patient-centered outcomes such as live birth [5]. These uncertainties persist despite rising clinical interest and the incorporation of SDF testing into recent American Urological Association and American Society for Reproductive Medicine (AUA/ASRM) (2024), European Association of Urology (EAU) (2025), and European Society of Human Reproduction and Embryology (ESHRE) (2022) guideline updates, underscoring the need for a structured, evidence-aligned approach [9,10,11]. Additionally, these factors highlight the necessity for a unified framework that integrates biological insights, assay characteristics, and guideline-based indications to optimize the use of OS and SDF testing in contemporary male infertility care.

This narrative review aims to: (i) delineate biological links between OS and SDF; (ii) summarize contemporary platforms for assessing seminal redox status and DNA integrity; (iii) map clinical contexts in which these measures refine evaluation and treatment; (iv) critically appraise recent guidelines and consensus statements; (v) identify evidence gaps and priorities for research and implementation; and (vi) offer a concise, decision-oriented framework, aligned with guideline indications, on when to measure OS alone, SDF alone, or both OS and SDF.

## 2. Biological Links Between Oxidative Stress and Sperm DNA Fragmentation

### 2.1. Physiological Versus Pathological Reactive Oxygen Species

ROS have a dual role in reproduction. At physiologic levels, redox signaling supports capacitation, hyperactivation, zona pellucida binding, and the acrosome reaction by modulating phosphorylation cascades and membrane remodeling primarily via controlled mitochondrial and nicotinamide adenine dinucleotide phosphate (NADPH)-oxidase-derived ROS [12,13]. When ROS generation exceeds seminal and intracytoplasmic antioxidant defenses, the chemistry turns deleterious: superoxide, hydrogen peroxide, hydroxyl radicals and related reactive nitrogen species drive lipid peroxidation, protein oxidation, and nucleic-acid damage, compromising motility, viability, and genomic integrity [8,14]. Thus, the same pathways that enable fertilization can, when unbuffered, produce structural injury, with sperm DNA as a principal target alongside membranes and key functional proteins [8,14].

### 2.2. Oxidative DNA Damage Cascade

Guanine oxidation to 8-hydroxy-2′-deoxyguanosine (8-OHdG) is a hallmark lesion recognized by 8-oxoguanine glycosylase-1 (OGG1), initiating base-excision repair (BER); OGG1 remains active in mature sperm, whereas completion of BER largely depends on oocyte-derived enzymes following fertilization [8,15]. In mature sperm, downstream BER steps (apurinic/apyrimidinic (AP) endonuclease incision, gap filling, ligation) are markedly curtailed due to loss of DNA-repair enzymes during spermiogenesis, allowing abasic sites and other intermediates to accumulate and evolve into single- and double-strand breaks that amplify SDF [16,17]. Oxidative stress also perturbs protamine disulfide cross-linking and generates lipid peroxidation–derived aldehydes (e.g., 4-hydroxynonenal, malondialdehyde) that form DNA and protein adducts and perpetuate mitochondrial ROS production [18,19]. Following fertilization, the oocyte assumes primary responsibility for repairing paternal lesions; this capacity declines with maternal age, modulating the clinical impact of SDF [20,21].

### 2.3. Sources of Reactive Oxygen Species in the Male Reproductive Tract

Endogenous and exogenous sources converge to elevate seminal ROS and promote SDF. Activated leukocytes, particularly neutrophils and macrophages in leukocytospermic semen, generate high fluxes of superoxide and hydrogen peroxide (and downstream oxidants such as hypochlorous acid via myeloperoxidase) that readily diffuse into sperm, overwhelming local antioxidants and causing oxidative DNA damage [22]. Male accessory gland infections and systemic inflammatory conditions, clinical varicocele, and metabolic derangements (e.g., obesity, metabolic syndrome) similarly increase ROS production in the reproductive tract, whereas dysfunctional sperm mitochondria contribute intrinsic oxidants [23,24,25,26,27,28,29].

**Figure 1:**
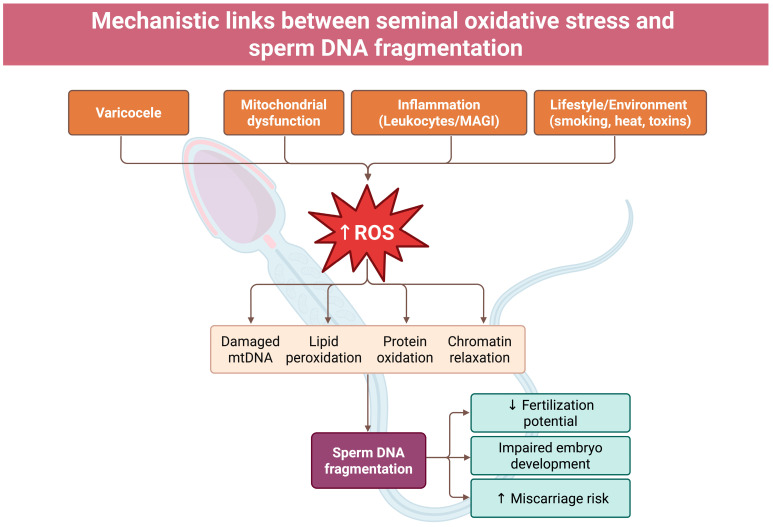
Mechanistic link between seminal oxidative stress and sperm DNA fragmentation. The figure illustrates how varicocele, mitochondrial dysfunction, leukocytes, and lifestyle/environmental factors contribute to increased reactive oxygen species (ROS). Excess ROS trigger DNA damage, lipid and protein oxidation, and chromatin relaxation, ultimately leading to elevated SDF and adverse reproductive outcomes. Abbreviations: MAGI, male accessory gland infection; ROS, reactive oxygen species. Created in BioRender. Created in BioRender. Kaltsas, A. (2026) https://BioRender.com/rb3mqjf.

External factors such as heat exposure, tobacco smoke, radiation, and industrial or environmental toxins (e.g., heavy metals, air pollutants) further exacerbate OS within the testes and epididymis, compounding the burden on sperm [28,30,31,32]. The net result is a spectrum of oxidative insults, ranging from lipid peroxidation of sperm membranes to oxidative base damage and single- or double-strand DNA breaks, that compromise sperm function and genetic integrity. The overall mechanistic cascade linking these upstream factors to genomic instability is summarized in Figure 1.

## 3. Assessment of Seminal Oxidative Stress

Evaluating OS in semen relies on biochemical assays that quantify ROS directly, infer global redox status, or measure oxidative damage by-products, complementing but differing from microscopy-based semen analysis in scope and interpretation [33]. Among available methods, direct ROS measurement and oxidation reduction potential (ORP) have broader clinical utility signals, whereas total antioxidant capacity (TAC) and reducing antioxidant power (FRAP) and oxidative adduct assays are supportive but not diagnostic. Single-cell fluorescence approaches using fluorescence microscopy or flow cytometry can further characterize oxidative heterogeneity and redox compartmentalization, including cytosolic and mitochondrial ROS, but currently remain adjunctive because of limited standardization and the absence of harmonized clinically validated cutoffs [34,35,36]. As summarized in Table 1, commonly used assays capture direct oxidants, global redox balance, antioxidant capacity, and oxidative injury, with distinct strengths, constraints, and clinical uses.

### 3.1. Direct Reactive Oxygen Species Measurement: Chemiluminescence

Chemiluminescence quantifies total ROS by detecting light emitted when probes—typically luminol or lucigenin—react with oxidants in neat semen or processed sperm fractions, with results commonly expressed as relative light units (RLU) per second per 10^6^ sperm (RLU/s/10^6^) [37,45]. Reference values derived in large cohorts that suggest cut-points around 10^2^ RLU/s/10^6^ sperm to discriminate fertile from infertile men have been proposed. However, no universally accepted diagnostic threshold exists because values depend heavily on laboratory protocol and probe chemistry [37,45]. Despite high analytical sensitivity, clinical uptake is constrained by the need for a luminometer, the absence of regulatory clearance for diagnostic use, and susceptibility to variability from probe concentration, pH/viscosity, incubation timing, and instrument calibration [46]. Inter- and intra-individual variability is considerable, and values fluctuate with time from ejaculation, abstinence interval, and intercurrent infection or inflammation, underscoring the need for standardized pre-analytics and cautious clinical interpretation [38]. A major limitation is that chemiluminescence quantifies total ROS and cannot differentiate ROS derived from sperm versus infiltrating leukocytes, necessitating parallel leukocyte assessment.

Single-cell fluorescent probe approaches by flow cytometry, such as 2′,7′ dichlorodihydrofluorescein diacetate (DCFDA) and dihydroethidium (DHE), provide complementary ROS readouts but remain research-oriented without unified clinical thresholds [46]. To address oxidant compartmentalization and cellular heterogeneity, contemporary single-cell panels often incorporate CellROX^®^ dyes, which are cell-permeant fluorogenic probes that report global cellular oxidative status, and MitoSOX™ Red, a mitochondria-targeted hydroethidine derivative designed to detect mitochondrial superoxide, thereby enabling a more granular oxidative phenotype than bulk chemiluminescence [35,47]. Probe interpretation requires a clear understanding of what each fluorescence readout most reliably captures and the conditions under which artifactual signals may occur. DCFDA is frequently used as an indicator of overall cellular oxidative status following intracellular de-esterification, but its fluorescence can be influenced by peroxidase activity and redox cycling reactions; therefore, it is best interpreted as a relative oxidative index rather than a species-specific ROS measurement [36]. DHE and MitoSOX are commonly used to interrogate superoxide-associated pathways in the cytosolic and mitochondrial compartments, respectively; however, hydroethidine-based probes can generate multiple oxidation products, and fluorescence intensity alone may not fully establish oxidant specificity, underscoring the need for appropriate controls and standardized acquisition parameters [48,49]. CellROX probes offer a practical, viability-compatible readout of oxidized viable sperm when combined with live–dead discrimination, thereby supporting clinically interpretable phenotyping [50].

A pragmatic clinical approach is to quantify and, when feasible, exclude leukocytes when genital tract inflammation is suspected, given their potential to dominate oxidative signals. Fluorescence-based ROS measurements are best interpreted as supportive evidence when they align with the semen phenotype, such as reduced motility or impaired vitality, and when they are concordant with complementary redox metrics including ORP or chemiluminescence. Serial reassessment after targeted optimization can strengthen interpretation by confirming the direction of change over time, while acknowledging that intra-individual variability and pre-analytical drift may be substantial. A mitochondrial-focused readout such as MitoSOX may provide mechanistic relevance when mitochondrial dysfunction is clinically plausible, particularly in asthenozoospermia. Results from MitoSOX assays have been associated with functional sperm parameters and assisted reproduction outcomes in selected cohorts [51].

Flow cytometry readouts are clinically interpretable only when sperm events are clearly separated from debris, aggregates, and leukocytes and when viability is explicitly gated. A minimal gating strategy begins with a forward- and side-scatter sperm gate with debris exclusion, followed by doublet discrimination using forward scatter area versus height or width to remove aggregates. Viability gating using propidium iodide (PI) or 7-aminoactinomycin D (7-AAD) should then restrict analysis to live sperm, while leukocyte identification or exclusion, using a marker such as cluster of differentiation 45 (CD45), is appropriate when leukocytospermia is present or suspected [52,53,54]. Reporting mean fluorescence intensity within the viable sperm gate, alongside the percentage of positive events, improves comparability across runs and reduces inflation from nonspecific staining in nonviable cells.

Specificity safeguards should be incorporated into every analytical run. A positive control, such as hydrogen peroxide–induced OS, confirms probe responsiveness and instrument performance, while unstained and single-stain controls enable appropriate compensation and background correction [38]. Because semen contains particulate debris and apoptotic sperm that may bind fluorogenic dyes nonspecifically, targeted fluorescence microscopy assessment can serve as a complementary validation step to confirm cellular localization of the signal and to exclude readouts driven predominantly by debris or non-sperm events [50].

### 3.2. Cell-Localized Superoxide Detection: Nitroblue Tetrazolium Assay

The nitroblue tetrazolium (NBT) assay gauges intracellular superoxide generation via reduction of NBT to insoluble formazan, enabling localization of ROS production within sperm (and any leukocytes) and semi-quantitative readout by absorbance of extracted formazan [55,56]. Proposed decision limits include >24 µg formazan per 10^7^ sperm as abnormal, aligning with higher ROS burdens observed in infertile men [56]. NBT can flag leukocyte-driven oxidative bursts and low-level granulocytic activity that confound semen redox assessments, aiding attribution of ROS sources in leukocytospermia [57]. Specificity is imperfect because non-superoxide reducing agents may also reduce NBT, and a lack of standard reference materials and harmonized protocols limits inter-laboratory comparability and routine clinical use [55,56,57]. Compared with chemiluminescence, the NBT assay demonstrates higher operator-dependent variability and lacks standardized colorimetric calibration curves.

### 3.3. Total Antioxidant Capacity: TAC and FRAP

TAC integrates enzymatic and non-enzymatic defense against oxidants by quantifying suppression of an induced oxidative reaction, with Trolox-equivalent antioxidant capacity (TEAC) formats reporting results in Trolox equivalents and demonstrating lower seminal TAC in infertile versus fertile men [42,58]. The FRAP assay measures the ability of seminal constituents to reduce Fe^3+^ to Fe^2+^, offering a complementary reductive capacity readout that can be implemented alongside chromogenic or chemiluminescent TAC methods [59]. Although TAC provides a global sense of redox buffering, method heterogeneity and substrate-specific differences impede cross-study comparability, and reported cutoffs (e.g., ~1.5–2.0 mM Trolox-equivalent) require local verification before clinical application [42]. However, TAC does not measure oxidants directly and often fails to correlate with clinically meaningful endpoints when used alone. Accordingly, TAC is most useful as an adjunct in suspected OS when direct ROS testing is unavailable or as a contextual marker alongside other redox metrics, rather than as a stand-alone diagnostic [59].

### 3.4. Redox Balance Metrics: Oxidation–Reduction Potential (MiOXSYS)

Oxidation-reduction potential (ORP) provides a composite “redox score” that integrates all oxidants and reductants, with the MiOXSYS system generating a static ORP (sORP) in millivolts normalized to sperm concentration (mV/10^6^ sperm/mL) for enhanced comparability across samples [60,61]. Across multiple cohorts, infertile men exhibit higher sORP than fertile controls, and thresholds around 1.3–1.5 mV/10^6^ sperm/mL have been proposed to discriminate abnormal redox status with acceptable diagnostic performance [41,62]. An additional analysis reported 1.36 mV/10^6^ sperm/mL as an optimal cut-point for abnormal semen quality, supporting the use of sORP as a screening metric in male infertility evaluations [40]. Practical advantages include rapid turnaround, small sample volume, and minimal sample processing, with studies noting that ORP may correlate with functional outcomes more robustly than isolated ROS or TAC indices in selected settings [63]. A 2024 meta-analysis pooling seven studies estimated a sensitivity near 81% and specificity near 66% for distinguishing infertile from fertile men, reinforcing the potential of ORP as an accessible first-line screen for seminal OS [64]. Methodological constraints remain: viscosity and incomplete liquefaction can influence sensor contact, and device guidance recommends testing within 60 min of ejaculation to limit drift, emphasizing strict pre-analytic control [65]. Importantly, sORP associates with sperm SDF on multivariable analyses, but leukocytospermia and extreme oligozoospermia may confound interpretation, arguing for integration with clinical context and complementary assays [66,67]. Despite encouraging multicenter data, conflicting evidence, including recent 2025 prospective findings, indicates that sORP alone lacks sufficient diagnostic reliability for comprehensive male infertility evaluation [64,68]. A critical limitation is that ORP reflects both sperm-derived and leukocyte-derived redox activity; without leukocyte quantification, abnormal ORP may be misattributed to intrinsic sperm dysfunction.

### 3.5. Biomarkers of Oxidative Damage: 8-OHdG and Lipid Peroxidation (MDA/TBARS)

Quantification of 8-hydroxy-2′-deoxyguanosine (8-OHdG) in sperm DNA provides lesion-specific evidence of oxidative base modification and correlates with elevated SDF and impaired reproductive outcomes, thereby offering mechanistic specificity at the expense of clinical availability and standardization [15]. Notably, 8-OHdG indicates oxidative base lesions but does not quantify strand breaks directly; therefore, elevations should be interpreted as mechanistic evidence rather than as a surrogate for SDF severity. Lipid peroxidation markers in seminal plasma, particularly malondialdehyde (MDA) measured by thiobarbituric acid reactive substances (TBARS) assays, reflect membrane oxidative injury and have been associated with reduced motility and higher SDF in infertile men relative to fertile controls, albeit with sensitivity to dietary and extrinsic influences and without consensus prognostic cut-points [44,69]. TBARS assays are known to overestimate MDA levels due to cross-reactivity with other aldehydes, limiting specificity. Given these constraints, 8-OHdG and MDA/TBARS serve best as corroborative indices of oxidative injury alongside global redox measures and SDF testing rather than as sole decision-making tools in routine practice [44,69].

To complement TBARS with a more cell-resolved assessment, C11-BODIPY581/591 has been employed as a ratiometric, spectral shift–based fluorescent probe of membrane lipid peroxidation that can be quantified by fluorescence microscopy or flow cytometry. This method captures lipid peroxidation at the single-cell level, including within viability-gated sperm populations, thereby improving interpretability compared with bulk TBARS measurements, while still requiring rigorous protocol standardization and laboratory-specific performance validation and thresholds [70,71].

### 3.6. Pre-Analytical Variables and Standardization Considerations

Uniform pre-analytics are critical across OS assays, including adherence to World Health Organization (WHO) collection standards, complete liquefaction, defined abstinence intervals, temperature control, and prompt testing or time-stamped storage to limit artifactual redox drift [46]. For chemiluminescence and NBT, assay performance depends on consistent probe concentrations, pH/viscosity, incubation timing, and instrument calibration, and laboratories should establish and regularly verify internal reference ranges and quality controls [38,46,55]. Testing within specified windows is particularly important for ORP, where device guidance recommends measurement within 60 min of ejaculation to minimize time-dependent changes in the semen redox milieu [65]. Centrifugation steps, especially high-speed or repeated centrifugation, can artificially increase ROS generation and thereby distort results of both chemiluminescence and ORP assays.

Mechanical handling represents an additional and frequently under-recognized source of analytical artifacts. Repeated pipetting, vigorous resuspension, and multiple wash steps can increase oxidative readouts by introducing shear stress and triggering redox-sensitive processes, thereby inflating apparent ROS signals even in the absence of genuine biological change [38,72]. Best practice therefore includes gentle and standardized sample handling with avoidance of repetitive pipetting, consistent timing from complete liquefaction to measurement, and explicit documentation of whether neat semen or washed sperm fractions were analyzed to support reliable intra-individual comparisons over serial testing.

Leukocytospermia inflates ROS and can distort ORP and chemiluminescent readouts; integrating leukocyte assessment and addressing infection or inflammation improves attribution of redox abnormalities to sperm versus infiltrating cells [22,25,30]. Therefore, all OS assays should include mandatory leukocyte quantification, either peroxidase staining or flow cytometry, to prevent diagnostic misclassification. Finally, documenting and, where feasible, standardizing abstinence intervals mitigates intra-individual variability in ROS and TAC measurements, and distinguishing neat semen from washed sperm protocols enhances comparability across serial tests and centers [38].

## 4. Diagnostic Evaluation of Sperm DNA Fragmentation

Over the past two decades, assays that quantify sperm DNA damage have moved from experimental use into the clinical evaluation of male infertility, with the principal platforms being the Sperm Chromatin Structure Assay (SCSA), terminal deoxynucleotidyl transferase dUTP nick-end labeling (TUNEL), the Comet assay, and the Sperm Chromatin Dispersion (SCD) test, each interrogating distinct biochemical facets of DNA integrity and therefore not being interchangeable in their readouts or thresholds [15,73]. Despite broad uptake, current SDF methods do not uniformly or independently predict assisted reproductive technology (ART) outcomes, and routine testing for all infertility evaluations remains debated [74,75,76]. Mechanistically, TUNEL and the alkaline Comet assay directly label or resolve DNA strand breaks, whereas SCSA and SCD infer damage from denaturation susceptibility or dispersion behavior and are therefore considered indirect measures [74]. In clinical practice, the usefulness of each assay depends not only on its mechanistic basis but also on local laboratory expertise, the clinical question being asked, and whether test results will directly influence management decisions.

### 4.1. Sperm Chromatin Structure Assay (SCSA)

SCSA assesses sperm DNA’s susceptibility to acid-induced denaturation with acridine orange, and high-throughput flow cytometry quantifies red (single-stranded) versus green (double-stranded) fluorescence across thousands of cells to derive the DNA Fragmentation Index (DFI) and a High DNA Stainability (HDS) metric of chromatin immaturity [15]. Robust standardization and internal controls support reproducible inter-laboratory results, underpinning its long-standing use in clinical and research settings and its frequent citation in guidelines; however, society guidelines consistently stress that SCSA thresholds are assay-specific and probabilistic rather than absolute [15,77]. Population data commonly categorize DFI <15% as low, 15–25% as intermediate, and >25–30% as elevated, with cut-offs applied probabilistically rather than absolutely [78]. Cohort and meta-analytic evidence indicate that DFI ≥25% is associated with lower pregnancy rates and higher miscarriage rates compared with DFI <15% for natural conception and intrauterine insemination (IUI), and DFI >50% is linked to poorer in vitro fertilization (IVF) outcomes [20]. Clinically, SCSA is particularly valuable when high-throughput, reproducible longitudinal monitoring is needed or when laboratory flow-cytometric capability makes it the most reliable locally available platform.

### 4.2. Terminal Deoxynucleotidyl Transferase dUTP Nick-End Labeling (TUNEL)

TUNEL directly labels free 3′-OH termini at DNA strand breaks via terminal deoxynucleotidyl transferase-mediated incorporation of tagged nucleotides, enabling quantification of the fraction of sperm with bona fide single- and double-strand breaks by flow cytometry or fluorescence microscopy [15]. Analytical nuances include variable nuclear decondensation steps to improve enzyme access in protamine-dense chromatin and the need to gate viable cells or apply vitality counterstains to avoid spurious positivity from membrane-compromised sperm [79,80]. Comparative studies indicate that TUNEL correlates strongly with fertility outcomes and, in some analyses, slightly outperforms SCSA for prediction of natural conception and IVF success, although protocols and thresholds differ among laboratories [76,81]. Because TUNEL conditions influence signal magnitude, there is no universal cutoff; nonetheless, many programs use ≈20% TUNEL-positive sperm as a commonly used decision threshold for high fragmentation when aligned with local validation [82,83]. Operationally, TUNEL is widely used in academic and high-complexity centers where fluorescence instrumentation and rigorous quality control are available, and it provides a complementary, mechanism-proximal readout alongside denaturation-based methods [84]. Because TUNEL directly measures strand breaks, many clinicians consider it the most mechanistically aligned with clinically relevant DNA damage; however, its interpretability requires careful adherence to technical parameters and locally validated reference ranges.

### 4.3. Single-Cell Gel Electrophoresis (Comet) Assay

The Comet assay (single-cell gel electrophoresis) embeds individual sperm in agarose and applies neutral or alkaline conditions to resolve primarily double-strand or both single- and double-strand breaks, respectively, with electrophoretic migration of fragmented DNA forming a fluorescent “tail” whose extent (e.g., tail length, tail DNA%, tail moment) quantifies per-cell damage [85]. The high sensitivity of Comet assay allows detection of low-level and lesion-specific damage, and results have been associated with impaired fertilization, embryo development, and pregnancy outcomes across multiple studies [85,86].

Despite these strengths, Comet remains constrained in clinical practice due to its manual nature, operator-dependent scoring, and variability across protocols and electrophoresis conditions [87,88]. Its strongest role remains in research or specialized reference laboratories, where it complements population-level flow cytometric assays by providing cell-resolved damage distributions [88]. In comparative contexts, alkaline Comet often shows high discrimination of infertile from fertile men, whereas neutral Comet is less informative for routine infertility workups [89]. More recent analyses suggest that mean Comet scores, together with distributions that quantify the proportions of sperm with high or low damage, add diagnostic value for male infertility and may improve prediction of IVF and intracytoplasmic sperm injection (ICSI) live-birth outcomes beyond binary cut-points [74]. Clinically, Comet results should be interpreted cautiously unless the laboratory has well-established internal standardization and extensive experience with the assay.

### 4.4. Sperm Chromatin Dispersion (SCD) Test

SCD (the “halo” test) induces controlled DNA denaturation and protein removal so that sperm with intact genomes form large halos of dispersed DNA loops, whereas fragmented genomes yield small or absent halos, permitting microscopic scoring of hundreds of cells for a percent-fragmented readout [90]. SCD correlates broadly with SCSA and TUNEL and differentiates fertile from infertile cohorts, with kit-based implementations facilitating practical adoption in laboratories lacking flow cytometry [90]. However, because SCD infers fragmentation from dispersion patterns rather than directly measuring strand breaks, interpretation can be confounded by nuclei with very tight packaging or protamination defects, which may limit halo formation independent of true fragmentation [90]. Clinical series demonstrate that higher percentages of no-halo sperm are associated with reduced reproductive potential, and observational data suggest the test’s utility in triaging couples for further evaluation or intervention [91]. As with other platforms, laboratories establish and verify local reference intervals, with ≳30% no-halo sperm commonly considered elevated in published cohorts using SCD kits [92]. For laboratories without flow-cytometric capacity, SCD provides a simple and scalable entry point into SDF testing, provided its limitations are recognized.

### 4.5. Comparative Performance and Assay Selection of SDF Tests in Clinical Practice

Head-to-head analysis within the same cohort shows that all major assays—TUNEL, SCSA, SCD, and alkaline Comet—distinguish infertile patients from fertile donors to varying degrees, with only neutral Comet failing to discriminate, and with correlations among assays being moderate, reflecting their measurement of partially overlapping biological constructs [89]. These moderate correlations underscore that each platform captures different aspects of chromatin instability, direct strand breaks, denaturation susceptibility, or halo-based dispersion, rather than interchangeable measures of a single underlying defect. Mechanistic studies link SDF to chromatin immaturity and apoptosis-related pathways, helping explain why TUNEL and alkaline Comet, which detect strand breaks directly, often align more closely with clinical endpoints than denaturation-based metrics, while SCSA remains the most standardized platform for multi-site use [93,94]. In practice, assay selection should be driven by the laboratory’s expertise and quality systems, the clinical question (screening versus mechanistic depth), and the need for throughput and standardization for longitudinal monitoring [84]. For example, SCSA offers unparalleled reproducibility for high-volume centers, whereas TUNEL provides direct lesion quantification when mechanistic fidelity is prioritized. For centers with robust flow cytometry and established protocols, SCSA and TUNEL provide complementary information; where microscopy-based workflows predominate, SCD offers accessible implementation with validated kit procedures [84]. Across all platforms, harmonized pre-analytics, stringent internal controls, and locally validated clinical decision thresholds are indispensable for maintaining interpretability over time and enabling meaningful comparison across centers [84]. Given inter-laboratory variability and heterogeneous study designs, professional guidance remains cautious about recommending universal, routine SDF testing for all patients, favoring targeted use where the result will change management [74,75,76]. Thus, real-world assay selection is best viewed not as identifying a universally superior platform, but as choosing the test that aligns operationally and clinically with the specific decision pathway being considered.

### 4.6. Ancillary and Emerging Approaches of SDF Testing

Older microscopy-based acridine orange tests provide qualitative denaturation assessments but lack the precision and standardization of flow cytometric SCSA, limiting their contemporary role to ancillary use where resources are constrained [95,96]. Histochemical stains such as aniline blue, toluidine blue and Chromomycin A3 (CMA3) index chromatin packaging defects rather than strand breaks and therefore serve as complementary markers of protamination status rather than primary measures of DNA fragmentation [95]. CMA3 is a fluorochrome-based stain used as an indirect marker of protamine deficiency and abnormal chromatin packaging [97]. It binds to GC-rich sites and competes with protamines, so increased CMA3 staining suggests incomplete protamination [97]. These stains may be useful adjuncts when assessing suspected chromatin remodeling abnormalities, but their findings should not be conflated with true DNA fragmentation indices.

Immunostaining for specific damage markers (e.g., γH2AX) and assays that quantify oxidative adducts (e.g., 8-oxoguanine labeling) remain research tools with potential to refine etiologic attribution of SDF in future clinical workflows [96]. Such lesion-specific approaches may eventually help distinguish oxidative from apoptotic or abortive-maturation pathways, allowing more tailored therapeutic decision-making. As these modalities mature, integration with standard SDF platforms may enhance mechanistic specificity, particularly when oxidative and non-oxidative pathways co-exist in the same ejaculate [96]. However, until analytical validation, reference ranges, and clinical utility are established, these should be interpreted cautiously and used primarily within research or specialized investigative settings.

### 4.7. Interpretation, Thresholds, and Reporting Standards of SDF

Interpretation is assay-specific, and identical percentage values carry different implications across platforms; for example, 28% by SCSA (DFI) generally reflects high fragmentation, whereas similar percentages on SCD or TUNEL occupy different risk strata depending on the laboratory’s validated cutoffs and protocols [82,83,92,98]. This inter-assay variability precludes universal cut-points and reinforces the need for laboratory-validated reference ranges that consider assay principle, pre-analytics, staining chemistry, and analytical sensitivity.

International consensus statements and practice guidelines provide context-specific thresholds and indications for SDF testing but emphasize that cutoffs are probabilistic and should not be used in isolation from the clinical picture [99,100]. As a realistic rule derived from pooled evidence, ≳25–30% fragmentation by the main assays is often considered abnormal and associated with adverse reproductive outcomes, while recognizing heterogeneity between platforms and centers [78,84]. For SCSA, >30% DFI is commonly termed severely elevated, 15–30% intermediate, and <15% low, whereas laboratories using TUNEL often adopt >20% as a high threshold based on local validation and published experience [82,83,101]. Several datasets also identify a practical DFI boundary around 25% on SCSA for reduced natural/IUI success, and report particularly unfavorable IVF outcomes when DFI exceeds ~50%, though centers should validate these ranges locally and interpret them alongside clinical context [102]. For SCD, thresholds around >30% no-halo sperm are frequently cited in studies associating fragmentation with reduced clinical pregnancy rates, though laboratories should verify these values in their own populations [92]. Reports should routinely specify the assay used, reference or decision thresholds, pre-analytical conditions, and quality-control metrics to facilitate interpretation and comparability across time and centers. Where possible, laboratories should include information about internal controls, coefficient of variation, gating strategy (for flow cytometry), and decondensation or lysis parameters, as these factors significantly influence measured SDF. Transparent reporting of these elements enhances reproducibility and supports appropriate clinical use in longitudinal monitoring and inter-laboratory benchmarking [84].

## 5. When to Order OS, SDF, or Both: A Clinical Decision Guide

The clinical value of OS and SDF testing lies in their complementarity: OS metrics characterize an injurious seminal milieu, whereas SDF quantifies the downstream genomic damage, and although correlated they are not redundant, so concurrent assessment refines diagnosis and guides mechanism-based intervention in ways not achievable with routine semen analysis alone [100,103]. A concise, decision-oriented summary of when to order OS, SDF, or both is provided in Table 2 below.

### 5.1. Genital Tract Infection

Genitourinary infection and subclinical inflammation—including male accessory gland infection—promote infiltration of white blood cells (leukocytes) into semen. These leukocytes generate high levels of ROS that overwhelm seminal antioxidant defenses, depress antioxidant capacity, and impair sperm function and DNA integrity, thereby increasing SDF [104,105]. Accordingly, targeted identification and treatment of leukocytospermia is a priority in mechanism-based care [22,25,30]. Because this pathway represents a potentially reversible source of oxidative and genomic injury, infection-related OS is among the clearest clinical indications for initial OS testing.

Because infection-related OS is potentially reversible, initial evaluation should include seminal OS testing (e.g., measurement of ROS or redox potential) to document this burden before attributing infertility to intrinsic sperm defects. Elevated oxidative markers confirm that infertility is at least partly due to infection/inflammation-driven oxidative damage [104,105]. Laboratory tools such as NBT/chemiluminescence assays and leukocyte quantification help attribute ROS production to infiltrating leukocytes versus sperm, thereby guiding antimicrobial and anti-inflammatory treatment rather than empirical antioxidant monotherapy [39,106]. Directed therapy which includes antibiotics for culture-positive infection, and anti-inflammatory agents, reduces ROS and can lower SDF in men with inflammatory semen profiles. This provides a reversible explanation for DNA damage in a subset of cases that might otherwise appear unexplained [22,25,30]. Post-treatment reassessment should include repeat OS testing to verify redox normalization. If fertility remains poor after the infection has resolved, SDF testing can be added to evaluate whether residual DNA damage persists. This stepwise process closes the loop on completes evaluation of an inflammation-driven pathway before escalating to ART-based solutions [22,25,30]. Persistently elevated SDF after successful infection control suggests either residual post-inflammatory injury or concurrent non-infectious etiologies and therefore justifies escalation to targeted interventions or ART rather than extended conservative management.

### 5.2. Microbiome Dysbiosis

Emerging evidence links imbalances in the male reproductive tract microbiome with elevated OS in semen. Certain bacteria prevalent during dysbiosis can induce ROS production or reduce antioxidant levels, creating a higher oxidative redox potential in semen [25,107]. This oxidative environment may impair sperm motility and DNA integrity, contributing to subfertility even without overt infection. Thus, if clinical or laboratory findings suggest microbial dysbiosis (abnormal semen culture or prostatitis-like symptoms without frank infection), a seminal OS test is recommended. High ROS or a low TAC may indicate that dysbiosis is causing oxidative sperm damage. Treatment involves correcting the microbiota imbalance (for example, targeted antibiotics or probiotics). After microbiome normalization, repeat OS testing should show improvement. If infertility continues despite improvement in oxidative markers, SDF testing should be added. This helps identify persistent DNA damage that may not resolve with microbiome correction and may require advanced assisted reproductive techniques [108].

### 5.3. Lifestyle and Environmental Modifiers of Seminal Oxidative Stress

Modifiable external factors such as cigarette smoking, chronic heat exposure (for example, frequent hot baths), air pollution, and severe psychological stress are established drivers of OS in semen [109]. These exposures increase the production of ROS in the testes and epididymis. This promotes lipid damage in sperm membranes and can create breaks in sperm DNA. For men with notable oxidative exposures, obtaining a baseline OS assessment such as ROS levels or ORP is useful. This establishes the current degree of redox imbalance and provides a reference point for later improvement [93].

Guideline-based first-line management is lifestyle optimization when oxidative or sperm DNA fragmentation indices are elevated [9,10]. Key measures include smoking cessation, weight control, avoidance of environmental toxins and excessive heat, regular exercise, a balanced diet, and stress reduction. These steps reduce OS in semen and can improve sperm DNA integrity over time [5]. Observational and translational data link smoking cessation and broader lifestyle change with lower SDF and improved motility, supporting their use as foundational therapy while specific drivers are addressed [110,111]. During attempts at natural conception or when banking/collecting for ART, shortening ejaculatory abstinence to ≤2 days and, in selected men, obtaining a second ejaculate approximately three hours after the first are low-cost adjuncts that reduce ejaculate SDF [112,113,114].

Antioxidant supplementation can be considered as a time-limited adjunct in men with clear OS signatures. A 2025 meta-analysis reported an absolute SDF reduction of approximately 4–5% after about three months of oral therapy, although individual responses vary [112]. In a 2024 randomized trial, antioxidants improved SDF and favorably shifted protamination (histone-to-protamine ratio), suggesting benefits that extend to chromatin packaging quality [115]. However, large randomized controlled trials (RCTs) have not consistently translated these biochemical gains into higher pregnancy or live-birth rates, indicating that indiscriminate antioxidant use is unwarranted [116,117]. Men with identifiable oxidative risk, such as smokers or those with elevated ORP or ROS, are more likely to benefit. Improvements are less predictable in men with no obvious oxidative driver [101].

Follow-up OS testing after lifestyle changes should confirm that the oxidative burden has decreased. If fertility remains poor even after oxidative markers normalize, SDF testing is appropriate. Persistent DNA fragmentation may reflect damage from earlier exposures and should prompt consideration of additional treatment or earlier use of assisted reproductive methods [118]. If SDF remains high despite improvement in OS, this pattern suggests that the damage may arise from non-oxidative causes. In such cases, evaluation for chromatin packaging defects or intrinsic testicular factors may be helpful.

### 5.4. Recurrent Pregnancy Loss

High SDF is consistently associated with recurrent pregnancy loss (RPL). Male partners of women with RPL exhibit significantly higher SDF indices than fertile controls, and paternal DNA damage is implicated in early gestational loss when oocyte repair capacity is exceeded [119,120,121,122]. Despite variations in RPL definitions across studies, a meta-analysis pooling 24 studies using SCSA (*n* = 7), SCD (*n* = 9), and TUNEL (*n* = 8) corroborated this association while noting small samples and heterogeneous inclusion criteria [123].

Accordingly, in couples with ≥2 miscarriages, an SDF assay is indicated even when routine semen parameters are normal; a high result offers a plausible mechanistic explanation for prior losses and provides prognostic information for future outcomes [122]. Identification of elevated SDF reclassifies a subset of “unexplained” RPL into a potentially correctable male-factor pathway and yields a measurable biomarker to monitor response before subsequent conception attempts [123,124].

Management should address modifiable contributors to OS such as silent genitourinary infection or inflammation, smoking, and thermal or chemical exposures. Clinicians should also consider varicocele repair when appropriate and implement structured lifestyle and antioxidant strategies with planned reassessment. During attempts at conception or during assisted reproduction, strict short ejaculatory abstinence can reduce the accumulation of DNA damage. ICSI with advanced sperm selection methods such as hyaluronic acid binding, high magnification selection, or microfluidic sorting may help enrich for sperm with intact DNA, although definitive live birth data are still emerging [125,126].

For men with persistently high ejaculate SDF despite optimization and particularly after failed IVF or ICSI, using testicular rather than ejaculated sperm for ICSI can be considered. Testicular sperm extraction with ICSI targets DNA damage at its source and is associated with improved outcomes. Studies and meta-analyses report higher clinical pregnancy and live birth rates and lower miscarriage rates in the same men when testicular sperm are used, although some variation exists across studies. Testicular sperm typically demonstrate substantially less fragmentation than paired ejaculates from the same individual (approximately an 80% relative reduction in DNA fragmentation index at retrieval) consistent with avoidance of epididymal/post-testicular OS [127,128].

After a structured trial of reversible measures, testicular sperm extraction (TESE) followed by ICSI can be considered for couples with unexplained infertility and persistently raised SDF after unsuccessful ART, with counseling that live-birth outcomes are under-reported in several series and that potential benefits must be balanced against the risks and costs of an invasive retrieval, particularly in otherwise normozoospermic or unexplained cases [128,129,130,131,132]. In men with cryptozoospermia or oligozoospermia, any advantage of testicular over ejaculated sperm has not been confirmed in large randomized studies; available data are heterogeneous and well-designed RCTs are still required to define indications and effect sizes [130,131,132]. Escalation to TESE-ICSI should therefore be individualized. It should incorporate center expertise, laboratory capability, and couple preferences. It should be reserved for situations in which ejaculate SDF remains above a clinically relevant threshold and the ART history is unfavorable despite optimization [132,133,134].

SDF testing is thus recommended in RPL; when SDF is elevated, aggressive intervention and the selective use of assisted reproduction, with sperm-selection strategies as appropriate, are often warranted to reduce the risk of further miscarriage [99]. SDF results also provide a clear framework for counseling and help identify couples who may benefit from earlier ART rather than prolonged expectant management.

### 5.5. Unexplained Infertility

A normal semen analysis does not guarantee normal sperm function, as 25–40% of infertile men with otherwise normal count, motility, and morphology exhibit elevated SDF [135]. Such occult DNA damage can impair fertilization and embryo development, explaining infertility classified as “unexplained” by standard tests [136]. Accordingly, in couples with unexplained infertility and a normal basic work-up, SDF testing is often recommended because it frequently uncovers a hidden male factor; platform-specific thresholds ≥25–30% are associated with lower fecundity, prolonged time-to-pregnancy, and higher miscarriage risk, thereby altering prognosis and counseling even when conventional parameters are within reference limits [78,84]. When SDF is high, it refines prognosis by predicting lower natural conception rates and a possibly higher risk of IVF failure or miscarriage, and it prompts targeted management [90]. Incorporating measures of OS (such as ORP) alongside SDF helps distinguish oxidant-driven damage from non-oxidative etiologies and personalize therapy toward treatable sources (e.g., inflammation, or lifestyle exposures), whereas a discordant pattern (high SDF with normal ORP) should trigger evaluation for chromatin-packaging defects or testicular factors not captured by redox assays [137].

In practice, demonstrating high SDF or an adverse redox profile facilitates shared decision-making about the timing of assisted reproduction, the length of trials of lifestyle and antioxidant strategies, and whether to escalate earlier to ICSI or to incorporate adjunct sperm-selection methods—particularly for couples with constrained reproductive timelines [138]. Clinically, targeted interventions include lifestyle optimization (e.g., smoking cessation and antioxidant use) and treatment of subtle inflammation when present; when SDF is markedly elevated, proceeding directly to ICSI or employing sperm-selection techniques may be preferable to prolonged natural attempts [99]. After medical or lifestyle intervention, repeat SDF testing provides an objective measure of response; if SDF normalizes, attention should be redirected to alternative causes of infertility, whereas persistently high indices support further targeted management or earlier use of assisted reproduction [99]. Persistent elevation of SDF despite normal oxidative markers suggests involvement of non-oxidative injury, and further evaluation of chromatin structure or testicular function may help refine management.

### 5.6. Repeated Assisted Reproductive Technology Failure

In couples with repeated ART failure which may present as several failed IVF or ICSI cycles, poor embryo development, or embryos of consistently low quality, an underlying defect in sperm DNA integrity is frequently implicated. High SDF correlates with impaired blastocyst development, lower implantation, and increased clinical miscarriage even when overt male-factor infertility is absent [139]; large contemporary cohorts indicate that SDF exerts its most consistent effect downstream of fertilization, with limited impact on fertilization rates but clear associations with compromised embryo development and higher miscarriage in both IVF and ICSI—an effect stronger in IVF yet still evident in ICSI, where oocyte repair cannot fully compensate [100,134].

Elevated DFI (≥30%) has been linked to higher miscarriage odds and, in some datasets, reduced blastocyst quality, increased day-5 arrest, and lower mean birth weight among liveborn offspring, extending SDF relevance into perinatal outcomes [140]. These observations suggest that SDF may influence not only embryo development but also certain perinatal outcomes. Population-level signals also suggest a possible association between paternal SDF and preeclampsia after ART, plausibly via placental effects of paternally derived DNA damage, though confirmation in prospective studies is required before practice change is warranted [141]. Meta-analytic evidence further shows that sperm DNA damage is associated with significantly reduced clinical pregnancy rates in IVF/ICSI cycles [81].

Accordingly, SDF testing is appropriate in the setting of repeated ART failure. Parallel assessment of OS, for example, ROS levels or oxidation reduction potential, is helpful when causes such as varicocele or lifestyle exposures are suspected. This can confirm a role for oxidative injury and guide targeted treatment, including antioxidants, varicocele repair, and lifestyle modification [142]. When SDF is elevated, management should aim both to reduce DNA damage and to limit its impact on ART outcomes. Simple measures include shorter abstinence before semen collection and careful adjustments in laboratory preparation to reduce additional OS.

Microfluidic sorting reduces centrifugation-related OS and achieves an ~10% absolute SDF reduction in the processed fraction compared to density gradients. Meta-analyses and single-center studies associate microfluidic sorting with higher fertilization, implantation, and clinical pregnancy rates, as well as improved blastocyst formation. Some cohorts also report higher rates of euploid embryos [143,144]. Hyaluronic-acid–based selection leverages physiologic binding, has been linked to improved live-birth outcomes among older couples in association with better sperm DNA quality, and represents a low-overhead adjunct in ICSI cycles where fragmentation is a concern [145,146]. Intracytoplasmic Morphologically Selected Sperm Injection (IMSI) may benefit couples with prior ICSI failure or pronounced male-factor defects, although operator dependence and heterogeneous protocols temper generalizability [119]. These methods do not correct the cause of DNA damage. They simply reduce the proportion of fragmented sperm used for injection. They are best applied when fragmentation has been clearly documented and conservative management is unlikely to be successful within the available reproductive timeframe [143,144,145,146].

For couples with persistent high ejaculate SDF despite optimization, particularly after unsuccessful IVF/ICSI, the use of surgically retrieved testicular sperm for ICSI (TESE-ICSI) may be considered. Testicular sperm generally demonstrate lower DNA fragmentation than ejaculated sperm, and in several studies have been associated with higher clinical pregnancy and live-birth rates and lower miscarriage, although outcomes vary across studies [131,143]. An umbrella review of systematic reviews/meta-analyses found that the association between SDF and ART outcomes is weak or nonsignificant and cautioned against making strong policy recommendations based on current data [147]. In practice, SDF-guided changes should therefore be framed within individualized, couple-centered counseling that integrates female age, embryo history, and center expertise, prioritizing interventions supported by current guidelines and expert consensus [9,10,100,148]. Ultimately, identifying and actively managing high SDF through treatment of underlying causes, careful laboratory practice, and selective use of sperm selection or testicular sperm is important in couples with unexplained ART failures. A clear pathway that includes diagnosis, targeted treatment, and timely escalation can improve ART outcomes and reduce repeated failed cycles [99].

### 5.7. Varicocele-Associated Infertility

Varicocele is a common cause of male infertility and creates a testicular environment that favors OS. As the clinical grade of the varicocele increases, levels of seminal ROS and SDF also rise, driven by scrotal hyperthermia, hypoxia, and increased mitochondrial oxidant production in the testis and epididymis [149,150]. Men with clinical varicocele typically exhibit elevated seminal ROS and higher SDF than men without varicocele [142]. In men with a palpable varicocele and borderline indications for repair, such as subfertility with only mild semen abnormalities, combined assessment of OS and SDF is very helpful. Markedly increased ROS or an abnormal ORP supports a diagnosis of varicocele-related oxidative injury [151]. An elevated SDF index indicates DNA damage in sperm that is likely to reduce fertility potential [142]. These findings often tilt the risk–benefit analysis toward varicocelectomy and help set expectations for the time course and magnitude of change [9,10].

Systematic reviews and meta-analyses show that varicocelectomy reduces SDF and lowers oxidative damage markers across multiple studies, including analyses in which men with higher baseline SDF experienced the greatest improvement [101,152]. Additional meta-analyses likewise show meaningful average reductions in SDF after repair [152,153]. These data support varicocelectomy as a treatment that can reduce both OS and DNA damage, especially when preoperative SDF is high. A practical clinical approach is to repair the varicocele, address lifestyle and other modifiable OS factors, and then reassess after about three to six months. Improvement in OS markers is often seen relatively early, sometimes within a few months, as the testicular environment stabilizes [151,154]. SDF usually improves more slowly as new spermatogenesis takes place, and many men show significant reductions by around six months [155].

**Figure 2:**
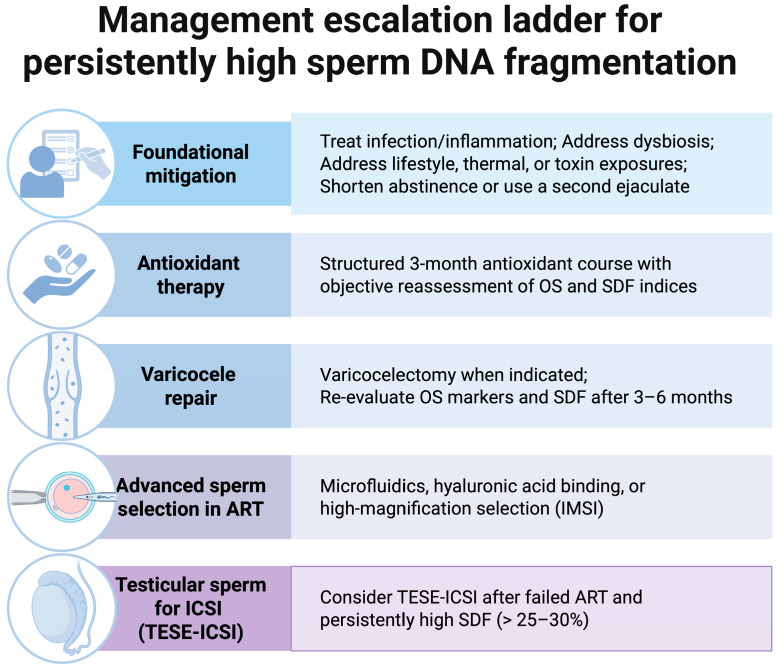
Management escalation ladder for persistently high sperm DNA fragmentation (SDF). Stepwise pathway: (1) foundational mitigation (treat infection/inflammation and dysbiosis; address lifestyle/thermal/toxin exposures; shorter abstinence or second ejaculate); (2) structured 3-month antioxidant therapy with objective reassessment of OS and SDF; (3) varicocele repair when indicated with re-evaluation at 3–6 months; (4) advanced sperm selection in ART (microfluidics, hyaluronic-acid binding, ±high-magnification/IMSI); (5) consider testicular sperm for ICSI (TESE-ICSI) after failed ART and persistently high SDF (>25–30%). Abbreviations: ART, assisted reproductive technology; IMSI, intracytoplasmic morphologically selected sperm injection; TESE-ICSI, testicular sperm extraction–intracytoplasmic sperm injection. Created in BioRender. Kaltsas, A. (2026) https://BioRender.com/6cp04tj.

Postoperative reassessment provides an objective basis for deciding between continued attempts at natural conception and progression to ART. If SDF remains elevated despite repair, couples should be counseled to consider assisted reproduction without unnecessary delay. In this setting, adjunctive sperm selection methods or, in selected cases, the use of testicular sperm for ICSI may be appropriate. If SDF normalizes or improves substantially, it is reasonable to continue expectant management or to use less invasive options [101,115]. Men with clinical varicocele and infertility who exhibit elevated SDF are reasonable candidates for repair, especially those with unexplained infertility, recurrent pregnancy loss, or prior ART failure [119,156]. Thus, combined OS and SDF testing identifies varicocele patients most likely to benefit from surgery, strengthens the rationale for intervention in equivocal cases, and supplies quantifiable endpoints to judge surgical success and guide subsequent fertility management [99]. A stepwise escalation pathway for persistent SDF is summarized in Figure 2.

### 5.8. Post-Intervention Monitoring (After Infection Treatment, Varicocelectomy, Lifestyle Changes, or Antioxidants)

In men who have undergone a therapeutic intervention to improve fertility, combined OS and SDF testing can objectively track recovery of sperm health. OS levels often improve quickly. For example, within a few weeks of antibiotic therapy for infection or after smoking cessation, seminal ROS markers frequently return to the normal range. This reflects rapid relief from an acute source of oxidative injury [99]. Sperm DNA fragmentation, on the other hand, may require a full spermatogenic cycle (~74 days) to show significant improvement, since it reflects the quality of newly produced sperm. Therefore, clinicians may first verify the early success of therapy by measuring OS. A normalization of ROS or a rise in TAC shortly after the intervention confirms that the seminal redox environment is now favorable. This is an important milestone, as persistent OS would continually damage developing sperm. Once sufficient time has passed for new sperm to be generated under improved conditions (usually 3–6 months), repeating the SDF assay will reveal whether DNA integrity has meaningfully improved [155]. A measurable decline in SDF is a positive sign. It indicates that fertility potential has increased and that attempts at natural conception or mild assisted reproduction, such as intrauterine insemination, may now have a better chance of success [99]. On the other hand, if SDF remains high despite corrected oxidative parameters, it suggests either an irreversible intrinsic issue or the need for additional measures. In such cases, assisted reproduction with techniques to mitigate sperm DNA damage (ICSI with testicular sperm or use of sperm selection devices) may be warranted rather than waiting longer [99].

## 6. Guidelines and Consensus Statements

Across recent updates, major societies converge on a selective, context-driven role for SDF testing. Routine screening of all infertile men is not recommended, but SDF is endorsed in specific scenarios—most consistently RPL, with broader adoption for unexplained infertility and repeated ART failure—while results should be interpreted with assay-specific thresholds and used to guide targeted interventions rather than in isolation [103].

### 6.1. AUA/ASRM (2024) Update

The 2024 amendment to the AUA/ASRM male infertility guideline does not endorse routine SDF testing in the initial evaluation, but supports its selective use in specific clinical scenarios, reflecting heterogeneity in methods, cut-offs, and mixed outcome data [9]. It explicitly recommends SDF assessment in couples with RPL, marking the first clear society-level endorsement of SDF in a defined clinical scenario [9]. When SDF is elevated, the guideline allows consideration of using testicular sperm for ICSI in non-azoospermic men as a therapeutic strategy within shared decision-making, while emphasizing that management should remain individualized and mechanism-informed [9]. These positions refine, but do not overturn, the foundational 2020–2021 guidance, which remains the basis for general male infertility evaluation and informs the selective integration of SDF testing [9,157].

### 6.2. EAU (2025) Update

The 2025 EAU Guideline on Male Sexual and Reproductive Health adopts a more expansive view of SDF testing than AUA/ASRM, recommending analysis in men from couples with RPL, in unexplained infertility despite a normal basic work-up, and after recurrent IVF/ICSI failure, based on Level 2a evidence that SDF provides actionable information for counseling and management [10]. The EAU advises addressing modifiable contributors (e.g., lifestyle, infection/inflammation, varicocele) when SDF is high and using test results to triage couples toward expectant management, optimized ART, or adjunctive selection strategies [10]. It underscores assay-specific interpretation and standardized pre-analytics when incorporating SDF into clinical pathways [10].

### 6.3. ESHRE Recurrent Pregnancy Loss Guideline (2022)

ESHRE’s RPL guideline (update 2022; published 2023) lists SDF assessment as an optional diagnostic add-on that may be considered in couples with RPL [11]. This reflects consistent associations between elevated paternal SDF and miscarriage yet acknowledging variability across assays and uncertainty about which interventions most improve live birth in this setting [11]. The recommendation is conditional, emphasizing integration with comprehensive couple-based evaluation and careful communication about probabilistic risk rather than deterministic causation [11].

### 6.4. Australasian Recurrent Pregnancy Loss Consensus (2024)

The Australasian RPL clinical management guideline (2024) recommends considering SDF testing in the work-up and provides a practical, stepwise algorithm to manage elevated SDF [158,159]. It prioritizes lifestyle optimization, treatment of infection/inflammation, consideration of varicocele repair, and time-limited antioxidant therapy, with escalation to ART incorporating advanced sperm selection or, where appropriate, the use of testicular sperm for ICSI when SDF remains persistently high [158,159]. The document operationalizes SDF-informed decision-making in RPL through stepwise pathways and re-testing, aligning with the broader shift toward mechanism-targeted male-factor management [158,159].

### 6.5. Sperm DNA Fragmentation Study Group (SFRAG) Guidelines (2021)

The Sperm DNA Fragmentation Study Group (SFRAG) practice guideline, developed by an international panel with expertise in reproductive urology and andrology, predated the most recent society updates but outlines broadly similar indications for SDF testing, including unexplained infertility, RPL, clinical varicocele, repeated ART failure, and fertility preservation settings [134]. It also supports re-testing after targeted interventions and selective consideration of testicular sperm for ICSI when ejaculate SDF remains persistently elevated [134].

### 6.6. Synthesis of Points of Consensus

Taken together, guidance published between 2022 and 2025 supports selective SDF testing—most consistently for couples with RPL—across regions and societies [9,10,11,158,159]. The EAU 2025 male infertility update further recommends SDF testing in men from couples with unexplained infertility after a normal basic work-up and in recurrent IVF/ICSI failure, emphasizing context-driven and assay-specific interpretation [10]. The AUA/ASRM 2024 amendment remains conservative about routine SDF testing in the initial male infertility evaluation, but explicitly endorses SDF assessment in RPL and allows consideration of testicular sperm for ICSI in selected non-azoospermic men with persistently elevated SDF within shared decision-making [9]. Across consensus and expert guidance, recurring themes include standardized pre-analytics, assay-specific reporting, locally validated thresholds, and using SDF results to inform mechanism-based interventions rather than as a stand-alone determinant [84,99,100,103,134,148]. The WHO laboratory manual (6th ed., 2021) describes DNA integrity and oxidative stress tests as extended/specialized assessments rather than routine semen analysis components [46]. Formal society guideline recommendations for seminal OS testing remain limited; therefore, OS testing is mainly guided by male oxidative stress infertility (MOSI)-style practice statements and expert consensus rather than society-level mandates.

## 7. Gaps in the Literature and Future Directions

Persistent variability in assay design, reporting, and interpretive thresholds continues to limit the translation of OS and SDF testing into uniform clinical pathways [138]. Closing these gaps will require coordinated standardization and improved etiologic attribution of damage. Progress will also depend on outcome-linked trials that include offspring-focused endpoints, evaluation of cost-effectiveness, and integration with couple-level prognostic models and emerging advanced diagnostics [160].

### 7.1. Standardization of Assays and Reporting

Despite decades of progress, universally harmonized protocols and population-based reference ranges are still lacking for key assays, particularly TUNEL, Comet, chemiluminescent ROS, TAC/FRAP, and ORP, so laboratories therefore rely on internally derived cut-offs that complicate cross-center comparison and meta-analysis [148,161,162,163]. Establishing common pre-analytics, calibration materials, and assay-specific reporting conventions analogous to WHO semen standards would improve interpretability and enable externally valid thresholds for “abnormal” SDF and seminal redox status in fertile versus infertile populations, while also clarifying contentious metrics such as ORP where recent data challenge earlier cut-offs [148,161,162,163]. Multi-site consortia focused on inter-laboratory validation and on mapping assay outputs to common clinical risk strata represent a feasible route to develop conversion tables across platforms until biomarker-agnostic probabilistic scales are validated [148,161,162,163].

### 7.2. Mechanistic Attribution of DNA Damage

Current tests quantify damage but rarely attribute it, and differentiating oxidative lesions from apoptosis-related or packaging-related breaks is essential for mechanism-based therapy and trial design [164]. Combining strand-break metrics with lesion-specific readouts such as 8-OHdG, alongside markers of chromatin remodeling (e.g., protamination indices), would help partition oxidative from non-oxidative SDF and align interventions with the predominant pathway [43,165]. Future diagnostics may employ multiplex panels integrating oxidative adducts, repair signatures, and chromatin-state markers to provide etiologic attribution rather than a unitary damage score, and, in future, could be complemented by epigenetic signatures that reflect cumulative redox exposure over spermatogenesis [43,164,165].

### 7.3. Offspring and Perinatal Health Outcomes

Beyond conception, contemporary datasets associate higher paternal SDF with increased miscarriage risk and, in some ART cohorts, with lower birth weight, raising the possibility of perinatal correlates beyond early gestation, although causality and long-term consequences remain uncertain [166]. Observational work and expert reviews also raise the possibility that sperm DNA damage and OS-linked lesions may influence placental function and offspring outcomes, framing paternal genome integrity as relevant to perinatal health rather than fertilization alone [129,141]. Parallel lines of evidence connect paternal age and the accrual of de novo mutations to adverse offspring risks, underscoring the need for prospective cohorts to clarify whether sperm damage burden and repair capacity at fertilization relate to long-term child health trajectories [167,168].

### 7.4. Couple-Based Prognostic Algorithms

Because oocyte repair capacity modulates the phenotypic expression of SDF, outcome prediction and triage should incorporate female age and reproductive reserve alongside male SDF/OS metrics to individualize recommendations about expectant management, timing of ART, and the intensity of male-directed interventions [9,10,84]. Guidelines already endorse selective SDF testing in RPL, unexplained infertility, and repeated ART failure, but decision support tools that integrate assay-specific thresholds with female factors and prior embryo history would better align treatment choices with prognosis and time horizons, as current risk calculators rarely incorporate SDF or OS in a couple-centric framework [9,10,84,113].

### 7.5. Cost-Effectiveness and Implementation Science

Economic evaluations remain sparse, and formal cost-effectiveness analyses of SDF/OS-guided pathways are limited by heterogeneity in tests, thresholds, and treatment strategies [9,169]. Even for promising platforms such as microfluidic sorting, health-economic evidence remains limited, and recent syntheses caution that widespread implementation should await definitive live-birth gains in prospective studies [143].

### 7.6. Male Aging and Germline Health

Multi-omics analyses implicate testicular niche remodeling, inflammation, and epigenetic drift in age-related declines in sperm quality, providing biologic links between aging, OS susceptibility, and heightened SDF, thereby pointing to senescence- and inflammation-targeted strategies that warrant prospective testing in older men [170]. Incorporating age-responsive endpoints, such as methylation clocks and small-RNA profiles, alongside SDF/OS measures could refine risk stratification and monitoring in advanced paternal age cohorts [170].

### 7.7. Raman Spectroscopy, AI-Assisted Selection, and Precision Diagnostics

Label-free Raman microspectroscopy can detect molecular signatures of nuclear damage and oxidative lesions at single-cell resolution, enabling non-destructive identification of sperm with intact genomes in experimental settings and remaining a proof-of-concept approach rather than a clinically validated selection tool, suggesting a path to real-time, mechanism-aware selection for ICSI if technical, validation, and cost barriers can be overcome [171,172,173,174]. Artificial intelligence (AI) applied to imaging and molecular data promises rapid, operator-independent triage of low-damage sperm and could be combined with microfluidic enrichment to reduce SDF in the inseminated cohort while preserving throughput, although these approaches remain pre-clinical and require rigorous clinical and regulatory evaluation before routine use [143,145,175].

### 7.8. In Vitro Spermatogenesis and In Vitro Gametogenesis

Emerging platforms—from organotypic slices and 3-D organoids to testis-on-a-chip and pluripotent-cell-derived gametogenesis—aim to restore fertility in severe male-factor contexts, yet remain at a preclinical stage and are not ready for routine clinical use, and clinical translation will require stringent release criteria that embed SDF, OS, and epigenomic integrity alongside genetic and functional benchmarks [176]. Ethical, regulatory, and quality-control frameworks specific to germline manipulation must evolve in tandem, with preclinical pipelines demonstrating stable genomic and epigenomic fidelity and long-term safety in relevant models before any application in ART can be contemplated [176].

### 7.9. Epigenetic Dimensions of Genome Integrity

Sperm epigenetic measures—including methylation-based “sperm epigenetic age”, locus-specific methylation signatures, histone-mark balance, and small-RNA cargo—associate with time-to-pregnancy, morphology, and chromatin compaction, and may integrate OS exposure over spermatogenesis to complement SDF in risk profiling [177,178]. Conceptual and empirical links between oxidative stress and epigenetic remodeling suggest that ameliorating OS could modulate epigenetic quality, with implications for embryo gene regulation and intergenerational health that merit prospective study [179,180,181]. Ultimately, panel-based diagnostics that pair strand-break, oxidative-lesion, and epigenetic integrity metrics may better capture the multidimensional nature of “genome health” than any single assay [179,180,181]. At present, however, epigenetic assays remain research tools without standardized thresholds or guideline endorsement for routine infertility evaluation, and their incorporation into clinical workflows should await analytical validation and outcome-linked studies.

### 7.10. Future Perspectives: Mitigation Strategies to Reduce OS-Related Sperm Damage

#### 7.10.1. Risk-Factor and Etiologic Mitigation

Future care models should position seminal OS as a treatable upstream phenotype that can be tracked and acted upon, rather than a static laboratory descriptor. In the near term, structured exposure mitigation (heat, tobacco, obesity/metabolic syndrome, air pollution, and occupational toxicants) can be operationalized as a “redox stewardship” bundle, with ROS or ORP/sORP (±TAC/FRAP) used for baseline stratification and objective follow-up rather than indiscriminate testing. Etiology-first pathways are likely to yield the highest clinical return: leukocytospermia or suspected male accessory gland infection/inflammation (MAGI) should prompt targeted microbiological evaluation and focused antimicrobial/anti-inflammatory management, with early re-assessment of redox status to document biologic response before escalation to longer empiric regimens. In OS/SDF-positive men with clinical varicocele, repair can be framed as a damage-mitigation intervention, supported by contemporary syntheses indicating reductions in SDF and oxidative lipid peroxidation markers after repair in selected cohorts [101].

#### 7.10.2. Precision Antioxidant and Redox-Targeted Therapeutics

Antioxidant use should transition from empiric supplementation to time-limited, OS-confirmed prescribing aligned with MOSI concepts, incorporating predefined stopping rules and monitoring to preserve physiologic redox signaling and limit the risk of reductive stress [4]. The neutral results of large, randomized trials in broadly defined infertility populations reinforce the need to test OS/SDF-guided algorithms (who to treat, for how long, and with what targets) rather than expanding universal supplementation [116,117]. Mid-term innovation should prioritize mechanism-aware approaches—mitochondria-focused antioxidants and redox modulators, rational combinations, and interventions paired to lesion type (oxidative adducts versus non-oxidative strand breaks)—but these require adequately powered trials with live birth as the primary endpoint and biomarker change as a secondary, supportive outcome.

#### 7.10.3. ART and Laboratory Mitigation

Laboratory workflows represent a controllable oxidative microenvironment and should increasingly be treated as an intervention point. Future protocols should explicitly minimize iatrogenic ROS generation by reducing centrifugation intensity, limiting time-to-processing, and standardizing temperature and oxygen exposure during handling. Microfluidic selection is a promising low-OS processing strategy that reproducibly lowers SDF in the selected fraction and is well suited for pragmatic trials embedded in routine IVF/ICSI workflows [143]. Physiologic sperm selection (e.g., hyaluronic acid binding) offers a low-overhead adjunct, with large datasets linking HA-based selection to improved outcomes in defined subgroups and to sperm DNA quality metrics [146]. For persistently high SDF despite upstream optimization—particularly after repeated ART failure—testicular sperm for ICSI may be considered in carefully selected non-azoospermic men, while acknowledging limitations of current evidence and the need for definitive randomized trials [132].

#### 7.10.4. Monitoring, Endpoints, and Implementation

A harmonized follow-up framework should be advanced: reassess OS within weeks after etiologic therapy and reassess SDF after approximately one spermatogenic cycle (≈74 days) or 3–6 months following varicocele repair, using the same assay platform to support longitudinal interpretation [34,182]. Implementation priorities include assay harmonization, cost-effectiveness analyses of OS/SDF-guided pathways, and prospective evaluation of device-based and AI-assisted selection strategies, with outcomes expanded beyond intermediate biomarkers to miscarriage, live birth, and perinatal health [99,183].

## 8. Conclusions

OS and SDF represent interconnected yet non-redundant dimensions of male-factor infertility, with oxidative injury, defective chromatin remodeling, and apoptotic dysregulation each contributing distinct routes to genomic compromise. The clinical expression of SDF is shaped not only by the burden and etiology of damage but also by the repair capacity of the oocyte, making identical SDF levels variably consequential across couples. Contemporary guidelines now converge on a selective, indication-based role for SDF assessment, particularly in RPL, unexplained infertility, and repeated ART failure, while underscoring assay-specific interpretation, standardized pre-analytics, and the importance of addressing modifiable contributors such as lifestyle, infection, inflammation, and clinical varicocele. Advanced assays measuring ROS or strand breaks can illuminate otherwise hidden mechanistic failure points but should complement, rather than replace, foundational semen evaluation; redox indices such as ORP remain investigational pending further validation. Progress in the field will require consistent laboratory protocols, standard reference ranges, and prospective trials linking test-guided interventions to live-birth and offspring-health outcomes. Emerging innovations, including lesion-specific oxidative markers, multiplex integrity panels, AI-assisted sperm selection, epigenetic signatures, and organoid-based gametogenesis, signal movement toward multidimensional assessment of sperm genome health, situating SDF and oxidative metrics within broader prognostic ecosystems. These developments refine our understanding of when and why OS and SDF matter clinically and lay the groundwork for more mechanistically informed, personalized, and outcome-driven approaches to male infertility care.

## Figures and Tables

**Table 1:** Assay landscape for seminal oxidative stress assessment.

Assay (Category)	What It Measures	Strengths	Limitations and Constraints	Typical Clinical Applications
Direct ROS (chemiluminescence) [37,38]	Total ROS in neat semen or washed sperm; RLU/s/10^6^ sperm	High analytical sensitivity; quantitative, measures oxidant burden directly	Requires luminometer; strict pre-analytics Does not directly distinguish sperm- vs. leukocyte-derived ROS; requires parallel leukocyte assessment Thresholds are protocol- and lab-dependent (probe, calibration)	Suspected oxidative stress Monitor response after targeted therapy
Cell-localized superoxide (NBT) [39]	Intracellular superoxide via NBT→formazan (semi-quantitative)	Helps localize ROS sources (sperm vs. leukocytes) Low-cost; feasible in routine labs	Operator-dependent; semi-quantitative Limited specificity; no standardized cutoffs	Leukocytospermia work-up; ROS source localization (sperm vs. leukocytes)
Redox balance (sORP; MiOXSYS) [40,41]	sORP (integrated oxidant-reductant balance); mV/10^6^ sperm/mL	Rapid; low volume; minimal processing Suitable for serial monitoring (same platform)	Affected by viscosity/incomplete liquefaction and time-to-test (strict testing window) Confounded by leukocytes and extreme oligozoospermia Not stand-alone; cutoffs are platform- and lab-dependent	Adjunct to assess/monitor redox imbalance alongside semen analysis ± SDF
Antioxidant capacity (TAC/FRAP) [42]	Global antioxidant capacity of seminal plasma (assay-dependent)	Context for antioxidant reserve Complements ROS/ORP measures	Indirect marker; does not measure oxidants Method heterogeneity; limited transferable cutoffs Influenced by diet, supplements, and inflammation	Adjunct marker; interpret with ROS/ORP and clinical context
Oxidative DNA damage biomarker (8-OHdG) [43]	8-OHdG in sperm DNA (oxidative base lesions)	Lesion-specific; supports oxidative attribution	Limited availability; specialized assays Complements SDF assays; does not quantify strand breaks	Specialized/research settings to corroborate an oxidative mechanism
Lipid peroxidation (MDA/TBARS) [44]	Seminal plasma lipid peroxidation (MDA via TBARS)	Links oxidative stress to membrane injury and motility/viability	Limited specificity (TBARS cross-reactivity); influenced by diet/external factors No universal cutoffs	Adjunct oxidative injury marker alongside ROS/ORP and SDF

Abbreviations: 8-OHdG, 8-hydroxy-2′-deoxyguanosine; FRAP, ferric reducing antioxidant power; MDA, malondialdehyde; MiOXSYS, MiOXSYS system; NBT, nitroblue tetrazolium; ORP, oxidation–reduction potential; ROS, reactive oxygen species; RLU, relative light units; SDF, sperm DNA fragmentation; sORP, static oxidation–reduction potential; TAC, total antioxidant capacity; TBARS, thiobarbituric acid-reactive substances.

**Table 2:** Targeted use of seminal oxidative stress (OS) and sperm DNA fragmentation (SDF) testing.

Clinical Scenario	Primary Test(s)	Rationale	Suggested Assay(s)	Recommended Action
Leukocytospermia/suspected genitourinary infection or inflammation (MAGI)	OS	Identify reversible oxidative burden driven by leukocytes/infection before labeling intrinsic sperm defects	ROS chemiluminescence (luminol/lucigenin) sORP (MiOXSYS) TAC/FRAP NBT	Treat infection/inflammation; re-test OS to confirm normalization. If infertility persists, add SDF to stratify prognosis
Microbiome dysbiosis (clinical/lab suspicion)	OS	Dysbiosis can elevate ROS and depress antioxidant capacity	ROS chemiluminescence sORP Leukocyte quantification	Correct dysbiosis; re-test OS.
Modifiable oxidative exposures (e.g., smoking, heat, toxins, lifestyle stresses)	OS	Establish baseline oxidative load and monitor response to risk-factor modification	ROS chemiluminescence sORP (screen/monitor)	Implement lifestyle/toxin mitigation; re-test OS
Recurrent pregnancy loss	SDF	SDF associates with miscarriage risk	TUNEL SCSA/SCD	If SDF is high; address modifiable sources (infection, varicocele, exposures), consider short abstinence, advanced sperm selection techniques; and discuss testicular sperm for ICSI in persistently high SDF cases
Unexplained infertility	SDF ± OS	Functional genomic damage may explain otherwise “normal” semen; SDF refines prognosis	TUNEL SCSA/SCD	If high SDF: targeted optimization (lifestyle, treat infection/varicocele), consider earlier ART with selection; monitor SDF after intervention
Repeated ART failure and/or poor embryo development	SDF ± OS	High SDF impacts embryo development and increases miscarriage rates; oxidative mechanism is suspected	TUNEL add OS panel if exposures/leukocytes/varicocele present	If high SDF: shorten abstinence, adopt microfluidic/HA-binding selection; consider testicular sperm for ICSI after failed cycles and persistent high SDF
Clinical varicocele	OS ± SDF	Seminal OS quantifies redox milieu; SDF quantifies genomic damage in varicocele patients	OS panel + TUNEL or SCSA/SCD	If OS/SDF elevated; consider varicocelectomy and re-test OS & SDF at ~3–6 months to guide expectant vs. ART strategy
Monitoring response after intervention	OS ± SDF	OS changes early with therapy; SDF reflects clinically meaningful improvement	Repeat same assays used at baseline for comparability	Use OS to verify early correction; use SDF to judge readiness for natural conception/IUI or need to escalate to ART

OS assays. Prefer direct ROS chemiluminescence where available. sORP (MiOXSYS) is practical for screening/monitoring but should not be the sole determinant of male fertility. TAC/FRAP are adjuncts only. SDF assays & thresholds. Prefer direct strand-break tests (TUNEL; alkaline Comet). SCSA/SCD are indirect but standardized/accessible. Use locally validated cut-offs; In practice, TUNEL ≈ ≥20% and SCSA (DFI) ≈ ≥25–30% often indicate clinically significant fragmentation (interpret probabilistically). Abbreviations: 8-OHdG, 8-hydroxy-2′-deoxyguanosine; ART, assisted reproductive technology; Comet, single-cell gel electrophoresis (Comet) assay; FRAP, ferric reducing antioxidant power; HA, hyaluronic acid; ICSI, intracytoplasmic sperm injection; IUI, intrauterine insemination; MAGI, male accessory gland infection/inflammation; NBT, nitroblue tetrazolium; ORP, oxidation–reduction potential; OS, oxidative stress; ROS, reactive oxygen species; RPL, recurrent pregnancy loss; SCD, Sperm Chromatin Dispersion (test); SCSA, Sperm Chromatin Structure Assay; SDF, sperm DNA fragmentation; sORP, static oxidation–reduction potential; TAC, total antioxidant capacity; TESE-ICSI, testicular sperm extraction–intracytoplasmic sperm injection; TUNEL, terminal deoxynucleotidyl transferase dUTP nick-end labeling.

## Data Availability

No new data were created or analyzed in this study. Data sharing is not applicable to this article.
